# Analysis of fractionalized Brinkman flow in the presence of diffusion effect

**DOI:** 10.1038/s41598-024-72785-2

**Published:** 2024-09-28

**Authors:** Shajar Abbas, Muhammad Ramzan, Inamullah Inam, Salman Saleem, Mudassar Nazar, Dilsora Abduvalieva, Hakim AL Garalleh

**Affiliations:** 1 Centre for Advanced Studies in Pure and Applied Mathematics, Bahauddin Zakariya University, Multan, Pakistan; 2 Department of Civil Engineering, Engineering Faculty, Laghman University, Mehtarlam, Afghanistan; 3 Department of Mathematics, College of Science, King Khalid University, Abha, 61413 Saudi Arabia; 4 Department of Mathematics and Information Technologies, Tashkent State Pedagogical University, Bunyodkor Avenue, 27, Tashkent, 100070 Uzbekistan; 5 Department of Mathematical Science, College of Engineering, University of Business and Technology, Jeddah, 21361 Saudi Arabia

**Keywords:** Diffusion-thermo, Heat transfer, Chemical reaction, Brinkman fluid, Mass transfer, Magnetic field, Engineering, Mathematics and computing

## Abstract

A vertical plate experiences a dynamic flow of fractionalized Brinkman fluid governed by fluctuating magnetic forces. This study considers heat absorption and diffusion-thermo effects. The novelty of model is the fractionalized Fourier’s and Fick’s laws. The problem is solved using the constant proportional Caputo derivative and Laplace transform method. The resulting non-dimensional equations for temperature, mass, and velocity fields are solved and compared visually. We explore the influence of various parameters like the fractional order, heat absorption/generation (Q), chemical reaction rate (R), and magnetic field strength (M) through informative graphs. Additionally, we contrast the velocity fields of fractionalized and regular fluids. The visualizations reveal that diffusion-thermo and mass Grashof number enhance fluid velocity, while chemical reaction and magnetic field tend to suppress it. For the interest of engineering, physical quantities such as Sherwood number, skin friction, and Nusselt number are computed. The present study satisfying all initial and boundary condition can be reduced to to previous published work which shows the validity of present work.

## Introduction

Due to variations in concentration and temperature, respectively, mass and heat transfer occur primarily in nature. Due to the overwhelming prevalence of these fluxes in nature, study in magnetohydrodynamics (MHD) is extremely important nowadays. The influence of a pressure gradient on a fluid was covered by Ahmed et al.^1^, whereas Ramzan et al.^2^ focused on the flow of MHD fluid in a channel with permeability effects. The soret effect above the surface with slip was investigated by Ref.^3^.

Physics, engineering, and applied mathematics are three fields where non-Newtonian fluids play a significant role. Its importance varies widely and may be found in many different fields, including plastic manufacture, biological fluids processing, and lubricant applications. Corn starch, paint, ketchup, colloids, custard, starch suspensions, melted butter, gels, toothpaste, shampoo, and blood are a few typical non-Newtonian fluid examples. The generation of entropy optimization with microorganism are analysed by Refs.^4–6^. Kumar et al.^7^ evaluated the radiative flux for riga with approach of stochastic. Computational reporting for solar collector in tube/disk with the influence of radiation are given by Refs.^8,9^.

MHD fluid has a significant range of uses in the areas of meteorology, petroleum industry, geophysics, energy generation, energy distillation, boundary layer control, polymer technology, astrophysics, and material processing, including drawing, extrusion, and wire for glass fiber. Authors in Ref.^10^ report on the fluid flow through a perpendicular plate. Chaudhary et al.^11^ have noted the impact of mass diffusion on a flow with porosity. The precise solution for the magnetic hydrodynamics flow through an inclined plate in the presence of porosity is determined by Sivaiah et al.^12^. Das et al.^13^ have found the answer to the problem of intermittent flow of a viscous fluid with porosity. Work on heat consumption was done by the author in Ref.^14^. The MHD flow across an inclined plate was covered by Ismail et al.^15^. The effects of mass transport in a channel were examined by Umavathi et al.^16^.

Convection flow in porous media has many uses, including heat exchangers for porous materials, heat flux in aerobic reaction, flows in soils, heat for fuel detritus, heat transfer associated with geothermal systems, solar power collectors, and thermal source in the domain of farming storage mechanisms.

Furthermore, there are several applications for convection flow in the presence of porosity, ground water hydrology, including heat exchangers im solid matrix, geothermal systems, oil refinement, the storage of radioactive waste, cooling systems, and cooling system. The solution of viscous fluid flow through a plate with heat radiation in magnetohydrodynamics was noted by Khan et al.^17^. Hartmann^18^ studied the MHD flow of a viscous fluid in a liquid metal with heat transfer. Murty et al.^19^ study the impact of heat transmission for solar cookers. Raja et al.^20^ apply a new method of solving a Casson fluid model. The impact of the stagnation point on Carreau fluid moving through a porous sheet was examined by Akbar et al.^21^.

Space technology greatly benefits from the understanding of how heat transfer affects different fluid flows. Kataria and Mittal^22^ explored the effect of heat radiation for a mathematical model of nanofluid via a plate. MHD fluid flow across a porous sheet was examined by Ref.^23^. The solution including the creation and absorption of heat from convective flow of nanofluid was examined by Abbasi et al.^24^. Heat absorption/absorption through a porous medium was used by Shehzad et al.^25^ to generate the Casson fluid solution. Analyzing the soret impact on MHD fluid convection flow across a plate was done by Sengupta and Ahmed^26^. Seth et al.^27^ discussed the solution for convection flow across a moving plate with non-uniform temperature was found.

In several branches of engineering and applied sciences, the mass diffusion discussion is empirically useful. The cooling of nuclear reactors, tabular reactors, the chemical industry, the petroleum industry, terracotta material mixtures, and the breakdown of stiff materials are all significantly impacted by this phenomenon. Thermal radiation and MHD fluid flow were examined by Khalid et al.^28^. Working with the Casson fluid was Kataria et al.^29^. Research on the effects of mass flux on fluid motion is conducted by Shah et al.^30^. Farhad et al.^31^ evaluated the accelerated velocity of fluid with slip. The authors^32^ spoke about the effects of Cuo-Water flow, while Mittal et al.^33^ focused on the Brownian motion of the Casson fluid. The stability of calculus derivatives was covered by Tran et al.^34^. Prabhakar fractional derivative effect on transport phenomena containing nanoparticles was examined by Asjad et al.^35^. Some helpful result uses^35–38^. Baithalu et al.^39^ observed the sensitivity analysis of heat flux with the technique of surface response. Thumma et al.^40^ reported the presence and absence of multislip conditions for the velocity of fluid. The methods of surface response, ANOVA test, and analysis of regression for the constitutive equation of momentum, energy, and mass diffusion are used by Refs.^41–44^

The fractionalized fluid MHD flow model via a plate is taken into consideration. The effects of heat absorption and diffusion thermodynamics are also taken into consideration. First, non-dimensionalization of the governing equations was done, and they were then semi-analytically solved. After obtaining the findings, a graphic analysis is done for the temperature profile, concentration profile, and velocity profile. Different graphs for the various parameters utilized in the flow model are displayed and explained.

## Novelty of current research

The novelty of present model are to answer the below question.

1. How is the temperature contour affected by significant values of *Du*?
2. How is the behavior of velocity profiles for different values of B and Gr?
3. What are the belongings on the concentration outline when the Schmidth number attain large values?
4. What is physics behind the result of multiple value of *fractionalparameter* for velocity and temperature of the fluid?

## Physical model

The flow of a Brinkman fluid over a vertical plate is examined here which is contained inside the region of $x_{2}^{\circ }$ > 0, where $x_{2}^{\circ }$ is a coordinate that has been measured perpendicular to the plate. At initial rest, both the liquid and plate have a constant temperature of $T_{\infty }^{\circ }$ at constant $t_{2}^{\circ } =0$. The heat and mass of the plate is increased simultaneously to $(T_{w}^{\circ }+T_{\infty }^{\circ })t_{2}^{\circ }/t_{2}^{\circ }+T_{\infty }^{\circ }$ when $t_{2}^{\circ }\le t_{2}^{\circ }$ and $(C_{w}^{\circ }+C_{\infty }^{\circ })t_{2}^{\circ }/t_{2}^{\circ }+C_{\infty }^{\circ }$ when $t_{2}^{\circ }\le t_{2}^{\circ }$ and $C_{w}^{\circ }$ for $t_{2}^{\circ }> t_{2}^{\circ }$ respectively as shown in Fig. 14a. Starting at a constant velocity of $Ue^{a^{\circ }t_{2}^{\circ }}$, the plate moves in the plane. linear momentum equation is^30^:

1 $$\begin{aligned} & \frac{\partial u_{0}(x_{2}^{\circ },t_{2}^{\circ })}{\partial t_{2}^{\circ }}+Bu_{0}+\frac{-\partial \tau }{\partial x_{0}^{*}}= g\beta _{T^{\circ }} (T^{\circ }-T_{\infty }^{\circ })\nonumber \\ & \quad -\frac{\sigma \beta _{0}^{2}u_{0}(x_{2}^{\circ },t_{2}^{\circ })}{\rho }-\frac{\mu u_{0}(x_{2}^{\circ },t_{2}^{\circ })}{\rho K_{2}}+ g\beta _{C^{\circ }} (C^{\circ }-C_{\infty }^{\circ }), \end{aligned}$$

shear stress $\tau$ is

2 $$\begin{aligned} \tau =\frac{\nu \partial u_{0}}{\partial x_{2}^{\circ }}, \end{aligned}$$

thermal equation is

3 $$\begin{aligned} \frac{\partial T^{\circ }}{\partial t_{2}^{\circ }}=- \frac{1}{\rho C_{p}}\frac{\partial q_{0}}{\partial x_{0}^{*}}-Q_{0}(T^{\circ }-T_{\infty }^{\circ })-\frac{D_{0}K_{T}\rho }{C_{s}}\frac{\partial J_{0}(x_{2}^{\circ },t_{2}^{\circ })}{\partial x_{0}^{*}}. \end{aligned}$$

$$q_{0}$$ is

4 $$\begin{aligned} q_{0}=-\alpha _{0}\frac{\partial T^{\cdot }}{\partial x_{2}^{\circ }}. \end{aligned}$$

Mass equation is

5 $$\begin{aligned} \frac{\partial C^{\circ }}{\partial t_{2}^{\circ }}=-\frac{\partial J_{0}}{\partial x_{0}^{*}}-R_{0}(C^{\circ }-C_{\infty }^{\circ }). \end{aligned}$$

$$J_{0}$$ is

6 $$\begin{aligned} & J_{0}=-D_{m}\frac{\partial C^{\circ }}{\partial x_{2}^{\circ }}, \end{aligned}$$

7 $$\begin{aligned} & \text {for} \,\,\,\,t_{2}^{\circ }=0, \,\,\,\,\,u_{0}(x_{2}^{\circ },0)=0, \,\,\, T^{\circ }(x_{2}^{\circ }, 0)=T_{\infty }^{\circ },\,\,\, C^{\circ }(x_{2}^{\circ }, 0)=C_{\infty }^{\circ }, \,\, \end{aligned}$$

8 $$\begin{aligned} & \text {for} \,\,\,x_{2}^{\circ }=0, \,\,\,\, u_{0}(x_{2}^{\circ },t_{2}^{\circ })=U f(t_{2}^{\circ }), \,\,\,T^{\circ }(x_{2}^{\circ },t_{2}^{\circ })=\left\{ \begin{array}{ll} T_{\infty }^{\circ }+\frac{(T_{w}^{\circ }-T_{\infty }^{\circ })t_{2}^{\circ }}{t_{0}}, & {0<t_{2}^{\circ }\le t_{0};} \\ T_{w}^{\circ }, & {t_{2}^{\circ }>t_{0},} \end{array} \right. \nonumber \\ & \quad C^{\circ }(x_{2}^{\circ },t_{2}^{\circ })=\left\{ \begin{array}{ll} C_{\infty }^{\circ }+\frac{(C_{w}^{\circ }-C_{\infty }^{\circ })t_{2}^{\circ }}{t_{0}}, & {0<t_{2}^{\circ }\le t_{0};} \\ C_{w}^{\circ }, & {t_{2}^{\circ }>t_{0},} \end{array} \right. , \end{aligned}$$

9 $$\begin{aligned} & \text {for} \,\,\,\,\,x_{2}^{\circ }\rightarrow \infty ,\,\,\,C^{\circ }(x_{2}^{\circ },t_{2}^{\circ })\rightarrow 0,\,\,\,\, T^{\circ }(x_{2}^{\circ },t_{2}^{\circ })\rightarrow 0,\,\,\, u_{0}(x_{2}^{\circ },t_{2}^{\circ })\rightarrow 0. \end{aligned}$$

## Generalization of problem

Non-dimensional quantities are

10 $$\begin{aligned} & x=\frac{Ux_{2}^{\circ }}{\nu },\,\,\,\,\,\,\,\, t=\frac{U^{2}t_{2}^{\circ }}{\nu },\,\,\,\,\,\,\,\, T=\frac{T^{\circ }-T_{\infty }^{\circ }}{T_{w}^{\circ }-T_{\infty }^{\circ }},\,\,\,\,\,\,\, u=\frac{u_{2}^{\circ }}{U}, \,\,\,\,\,\, \text {Gr}=\frac{g\nu \beta _{T^{\circ }}(T_{w}^{\circ }-T_{\infty }^{\circ })}{U^{3}},\nonumber \\ & \quad M=\frac{\beta _{0}^{2}\sigma }{\rho \text {Gm}=\frac{g\nu \beta _{C^{\circ }}(C_{w}^{\circ }-C_{\infty }^{\circ })}{U^{3}}\,\, U^{2}},\,\,\,\,\,\,Q=\frac{Q_{0}\nu }{U^{2}}, \,\,\,R=\frac{R_{0}\nu }{U^{2}},\,\,\,\,\,C=\bigg [\frac{C_{w}^{\circ }-C_{\infty }^{\circ }}{C^{\circ }-C_{\infty }^{\circ }}\bigg ]^{-1}. \end{aligned}$$

Generalization of Eq. (2) by Ref.^45^ is

11 $$\begin{aligned} \tau =L_{\beta } D_{t}^{\beta }\frac{\partial u}{\partial t}, \,\,\,\,\,\,\,0<\beta \le 1. \end{aligned}$$

where $1=L_{\beta }$ for $\beta \rightarrow 1$. use Eq. (10) and Eq. (2) into Eq. (1)

12 $$\begin{aligned} \bigg (\frac{\partial }{\partial t}+B+M+\frac{1}{K}\bigg )u(x,t)= & \text {Gr}T(x,t)+L_{\beta }\frac{\partial }{\partial x}\bigg [ D_{t}^{\beta } \frac{\partial u}{\partial x}\bigg ]+ \text {Gm}C(x,t). \end{aligned}$$

Equation (4) is generalized by Refs.^46,47^

13 $$\begin{aligned} -K_{\gamma }D_{t}^{\gamma }\frac{\partial T}{\partial x}=q_{1},\,\,\,\,\,\,0<\gamma \le 1. \end{aligned}$$

Use Fick’s Law on Eq. (6) as

14 $$\begin{aligned} J_{1}+D_{\alpha }D_{t}^{\alpha }\frac{\partial C(x,t)}{\partial x}=0,\,\,\,\,\,\,\,0<\alpha \le 1. \end{aligned}$$

Put Eqs. (13) and (14) in Eq. (3)

15 $$\begin{aligned} QT+\frac{\partial T}{\partial t}= \text {Pr}^{-1} \frac{\partial }{\partial x}\bigg [ D_{t}^{\gamma }\frac{\partial T}{\partial x}\bigg ]+ \frac{\partial }{\partial x}\bigg [ D_{t}^{\alpha }\frac{\partial C}{\partial x}\bigg ]Du, \end{aligned}$$

where $$\text {Pr}=\frac{\nu \rho C_{p}}{K_{\gamma }}$$.

Use Eq. (14) in Eq. (5)

16 $$\begin{aligned} \text {Sc}\bigg (\frac{\partial }{\partial t}+R\bigg )C= \frac{\partial }{\partial x}\bigg [ D_{t}^{\alpha }\frac{\partial C }{\partial x}\bigg ], \end{aligned}$$

where $$\text {Sc}=\frac{\nu }{D_{\alpha }}$$ or

17 $$\begin{aligned} \bigg (\frac{\partial }{\partial t}\frac{1}{K}+B+M\bigg )u(x,t)= & L_{\beta } D_{t}^{\beta } \frac{\partial ^{2} u}{\partial x^{2}}+ \text {Gr}T+\text {Gm}C, \end{aligned}$$

18 $$\begin{aligned} \frac{\partial T}{\partial t}= & \text {Pr}^{-1} D_{t}^{\gamma }\frac{\partial ^{2} T}{\partial x^{2}}-Q T+Du \bigg [ D_{t}^{\alpha }\frac{\partial ^{2} C}{\partial x^{2}}\bigg ], \end{aligned}$$

19 $$\begin{aligned} \frac{\partial C}{\partial t}= & \text {Sc}^{-1} D_{t}^{\alpha }\frac{\partial C^{2} (x,t)}{\partial x^{2}}-R C(x,t). \end{aligned}$$

With the following conditions

20 $$\begin{aligned} & \text {for}\,\,\,\,t=0, \,\,\,u(x,t)= T(x,t)=C(x,t)=0,, \end{aligned}$$

21 $$\begin{aligned} & \text {for} \,\,\, \,\,\,x=0, \,\,\, u(x,t)= e^{at},\,\, T(x,t)=C(x,t)=\left\{ \begin{array}{ll} t, & {0<t\le 1;} \\ 1, & {t>1} \end{array} \right. , \end{aligned}$$

22 $$\begin{aligned} & \text {for} \,\,\,\,\,\, x\rightarrow \infty , \,\,\,\,\,T(x,t)\rightarrow 0, \,\,\,u(x,t)\rightarrow 0\,\,\,\,\,C(x,t)\rightarrow 0. \end{aligned}$$

Fractional derivative is

23 $$\begin{aligned} D_{t}^{\eta }f(t)= \frac{1}{\Gamma (1-\eta )}\int _{0}^{t}\bigg (K_{1}(\eta )f(\tau )+K_{0}(\eta )f'(\eta )\bigg )(t-\tau )^{-\eta }d\tau . \end{aligned}$$

## Model’s solution

### Distribution of mass

Equation (19) is transformed using Laplace method to get

24 $$\begin{aligned} s{\bar{C}}= \text {Sc}^{-1}\bigg (K_{0}(\alpha )+\frac{K_{1}(\alpha )}{s}\bigg )s^{\alpha }\frac{\partial ^{2}{\bar{C}}}{\partial x^{2}}-\text {R}{\bar{C}}, \end{aligned}$$

satisfy

25 $$\begin{aligned} {\bar{C}}(x,s)=0 \,\,\,\text {and} \,\,\, \bigg (s^{-2}-e^{-s}s^{-2}\bigg )={\bar{C}}(0,s). \end{aligned}$$

Equation (25) is used in Eq. (24) as

26 $$\begin{aligned} {\bar{C}}=s^{-2}\bigg (1-e^{-s}\bigg )e^{-x\sqrt{\frac{(s+R)\text {Sc}}{\bigg (\frac{K_{1}(\alpha )}{s}+K_{0}(\alpha )\bigg )s^{\alpha }}}}. \end{aligned}$$

Equation (26) is difficult to solve analytically. we used algorithm^48,49^ for taking inverse.

### Temperature field

Equation (18) is transformed using Laplace method to get

27 $$\begin{aligned} s\text {Pr}{\bar{T}}(x,s) & = \bigg (+K_{0}(\gamma )+\frac{K_{1}(\gamma )}{s}\bigg )s^{\gamma }\frac{\partial ^{2}{\bar{T}}(x,s)}{\partial x^{2}}-\text {Q}\text {Pr}{\bar{T}}(x,s)\nonumber \\ & + \quad Du\text {Pr}\bigg (+K_{0}(\alpha )+\frac{K_{1}(\alpha )}{s}\bigg )s^{\alpha }\frac{\partial ^{2}{\bar{C}}(x,s)}{\partial x^{2}}, \end{aligned}$$

with

28 $$\begin{aligned} {\bar{T}}(x,s)=0 \,\,\, \text {and}\,\,\,\,\bigg (1-e^{-s}\bigg )s^{-2}={\bar{T}}(0,s). \end{aligned}$$

Equation (28) is used in Eq. (27) as

29 $$\begin{aligned} {\bar{T}}(x,s) & =\frac{1-e^{-s}}{s^{2}}e^{-x\sqrt{\frac{(s+Q)\text {Pr}}{\bigg (\frac{K_{1}(\gamma )}{s}+K_{0}(\gamma )\bigg )s^{\gamma }}}}+ \frac{(1-e^{-s})s^{-2}\text {Sc}(s+R)Du\text {Pr}}{ \frac{\bigg (s+R)(\frac{K_{1}(\gamma )}{s}+K_{0}(\gamma )\bigg )s^{\gamma }\text {Sc}}{\bigg (\frac{K_{1}(\alpha )}{s}+K_{0}(\alpha )\bigg )s^{\alpha }}- (s+Q)\text {Pr}}\nonumber \\ & \quad \times \bigg ( e^{-x\sqrt{\frac{(s+Q)\text {Pr}}{\bigg (\frac{K_{1}(\gamma )}{s}+K_{0}(\gamma )\bigg )s^{\gamma }}}}- e^{-x\sqrt{\frac{(s+R)\text {Sc}}{\bigg (\frac{K_{1}(\alpha )}{s}+K_{0}(\alpha )\bigg )s^{\alpha }}}}\bigg ), \end{aligned}$$

for $\beta =\alpha$, Rewrite Eq. (29) is

30 $$\begin{aligned} {\bar{T}}(x,s) & =\frac{1-e^{-s}}{s^{2}}e^{-x\sqrt{\frac{(\text {Pr}s+\text {Pr}Q)}{\bigg [\frac{K_{1}(\alpha )}{s}+K_{0}(\alpha )\bigg ]s^{\alpha }}}}+ \bigg [\frac{s^{-2}(Du-Due^{-s})\text {Sc}(s\text {Sc}\text {Pr}+R\text {Sc}\text {Pr})}{(\text {Sc}s+\text {Sc}R)- (s+Q)\text {Pr}}\bigg ]\nonumber \\ & \quad \times \bigg [ e^{-x\sqrt{\frac{s\text {Pr}+Q\text {Pr}}{\bigg [\frac{K_{1}(\alpha )}{s}+K_{0}(\alpha )\bigg ]s^{\alpha }}}}- e^{-x\sqrt{\frac{s\text {Sc}+R\text {Sc}}{\bigg [\frac{K_{1}(\alpha )}{s}+K_{0}(\alpha )\bigg ]s^{\alpha }}}}\bigg ]. \end{aligned}$$

The inverse of Eq. (30) is taken with algorithm^48,49^.

### Velocity field

Equation (17) is transformed using Laplace method to get

31 $$\begin{aligned} {\bar{u}}(x,s)(M+s+K^{-1}+B)= & L_{\beta }\bigg (\frac{K_{1}(\beta )}{s}+K_{0}(\beta )\bigg )s^{\beta }\frac{\partial ^{2}{\bar{u}}(x,s)}{\partial x^{2}}\nonumber \\ & + \text {Gr}{\bar{T}}(x,s) +\text {Gm}{\bar{C}}, \end{aligned}$$

with

32 $$\begin{aligned} {\bar{u}}(0,s)=\frac{1}{s-a},\,\,\,\,\,\,\,{\bar{u}}(x,s)=0. \end{aligned}$$

Equation (32) is used in Eq. (31) as

33 $$\begin{aligned} {\bar{u}} & =\frac{1}{s-a}e^{-x\sqrt{\bigg [\frac{(M+B+\frac{1}{K}+s)}{L_{\beta }\bigg [\frac{K_{1}(\beta )}{s}+K_{0}(\beta )\bigg ]s^{\beta }}\bigg ]}}\nonumber \\ & \quad + \bigg [\frac{\frac{\text {Gr}s^{-1+\beta }}{\bigg (\frac{K_{1}\beta )}{s}-K_{0}(\beta )\bigg )}}{ \frac{(\text {Pr}s+Q\text {Pr})}{\bigg (\frac{K_{1}(\gamma )}{s}-K_{0}(\gamma )\bigg )s^{\gamma }}- \frac{(M+\frac{1}{K}+B+s)}{L_{\beta }\bigg (\frac{K_{1}(\beta )}{s}+K_{0}(\beta )\bigg )s^{\beta }}}\bigg ]\nonumber \\ & \quad \times \bigg [\frac{1-e^{-s}}{s^{2}}+\frac{\frac{(1-e^{-s})Du\text {Sc}\text {Pr}(s+R)}{s^{2}\bigg (K_{0}(\alpha )+\frac{K_{1}(\alpha )}{s}\bigg )s^{\alpha }}}{\frac{(s+R)\text {Sc}}{\bigg (K_{0}(\alpha )+\frac{K_{1}(\alpha )}{s}\bigg )s^{\alpha }}- \frac{(s+Q)\text {Pr}}{\bigg (\frac{K_{1}(\gamma )}{s}+K_{0}(\gamma )\bigg )s^{\gamma }}}\bigg ] \nonumber \\ & \quad \times \bigg [e^{-x\sqrt{\bigg [\frac{(s+M+B+\frac{1}{K})}{L_{\beta }\bigg [\frac{K_{1}(\beta )}{s}+K_{0}(\beta )\bigg ]s^{\beta }}\bigg ]}}- e^{-x\sqrt{\bigg [\frac{(s+Q)\text {Pr}}{\bigg (\frac{K_{1}(\gamma )}{s}+K_{0}(\gamma )\bigg )s^{1-\gamma }}\bigg ]}}\bigg ]\nonumber \\ & \quad + \bigg [\frac{\frac{\text {1}}{\bigg (\frac{K_{1}(\beta )}{s}+K_{0}(\beta )\bigg )s^{\beta }}}{ \frac{(s+R)\text {Sc}}{\bigg (\frac{K_{1}(\alpha )}{s}+K_{0}(\alpha )\bigg )s^{\alpha }}- \frac{(s+M+B+K^{-1})}{L_{\beta }\bigg (\frac{K_{1}(\beta )}{s}+K_{0}(\beta )\bigg )s^{\beta }}}\bigg ] \nonumber \\ & \quad \times \bigg [\frac{\text {Gm}(1-e^{-s})}{s^{2}}-\frac{\frac{(1-e^{-s})Du\text {Gr}\text {Sc}\text {Pr}(s+R)}{s^{2}\bigg (\frac{K_{1}(\alpha )}{s}+K_{0}(\alpha )\bigg )s^{\alpha }}}{\frac{(s+R)\text {Sc}}{\bigg (\frac{K_{1}(\alpha )}{s}+K_{0}(\alpha )\bigg )s^{\alpha }}- \frac{(s+Q)\text {Pr}}{\bigg (\frac{K_{1}(\gamma )}{s}+K_{0}(\gamma )\bigg )s^{\gamma }}}\bigg ] \nonumber \\ & \quad \times \bigg [e^{-x\sqrt{\bigg [\frac{(s+M+B+\frac{1}{K})}{L_{\beta }\bigg [\frac{K_{1}(\beta )}{s}+K_{0}(\beta )\bigg ]s^{\beta }}\bigg ]}}- e^{-x\sqrt{\bigg [\frac{(s+R)\text {Sc}}{\bigg (\frac{K_{1}(\alpha )}{s}+K_{0}(\alpha )\bigg )s^{\alpha }}\bigg ]}}\bigg ], \end{aligned}$$

34 $$\begin{aligned} {\bar{u}}(x,s) & =\frac{1}{s-a}e^{-x\sqrt{\bigg [\frac{(s+M+B+\frac{1}{K})}{L_{\beta }\bigg [\frac{K_{1}(\beta )}{s}+K_{0}(\beta )\bigg ]s^{\beta }}\bigg ]}}\nonumber \\ & \quad + \bigg [\frac{\text {Gr}}{ \frac{L_{\beta }\bigg (\frac{K_{1}(\beta )}{s}+K_{0}(\beta )\bigg )s^{\beta }(s+Q)\text {Pr}}{\bigg (\frac{K_{1}(\gamma )}{s}-K_{0}(\gamma )\bigg )s^{\gamma }}- (s+M+B+K^{-1})}\bigg ] \nonumber \\ & \quad \times \bigg [\frac{-e^{-s}+1}{s^{2}}+\frac{(1-e^{-s})(R+s)\text {Sc}Du\text {Pr}}{s^{2}[ (\text {Sc}s+R\text {Sc})- (\text {Pr}s+\text {Pr}Q)]}\bigg ] \nonumber \\ & \quad \times \bigg [e^{-x\sqrt{\bigg [\frac{(s+M+B+\frac{1}{K})}{L_{\beta }\bigg [\frac{K_{1}(\beta )}{s}+K_{0}(\beta )\bigg ]s^{\beta }}\bigg ]}}- e^{-x\sqrt{\bigg [\frac{(s+Q)\text {Pr}}{\bigg (\frac{K_{1}(\gamma )}{s}+K_{0}(\gamma )\bigg )s^{1-\gamma }}\bigg ]}}\bigg ] \nonumber \\ & \quad + \bigg [\frac{\text {1}}{ L_{\beta }s^{\beta }\bigg (\frac{K_{1}(\beta )}{s}+K_{0}(\beta )\bigg )\frac{(s+R)\text {Sc}}{\bigg (\frac{K_{1}(\alpha )}{s}+K_{0}(\alpha )\bigg )s^{\alpha }}- (s+M+B+K^{-1})}\bigg ] \nonumber \\ & \quad \times \bigg [\frac{(\text {Gm}-e^{-s}\text {Gm})}{s^{2}}-\frac{\frac{(1-e^{-s})(s+R)\text {Gr}Du\text {Sc}\text {Pr}}{s^{2}}}{(\text {Sc}s+R\text {Sc})- (\text {Pr}s+Q\text {Pr})}\bigg ] \nonumber \\ & \quad \times \bigg [e^{-x\sqrt{\bigg [\frac{(s+M+B+\frac{1}{K})}{L_{\beta }\bigg [\frac{K_{1}(\beta )}{s}+K_{0}(\beta )\bigg ]s^{\beta }}\bigg ]}}- e^{-x\sqrt{\bigg [\frac{(\text {Sc}s+R\text {Sc})}{\bigg (\frac{K_{1}(\alpha )}{s}+K_{0}(\alpha )\bigg )s^{\alpha }}\bigg ]}}\bigg ], \end{aligned}$$

for $\beta =\alpha =\gamma$, Rearrangement of Eq. (34) is

35 $$\begin{aligned} {\bar{u}} & = (s-a)^{-1}e^{-x\sqrt{\bigg [\frac{(s+\frac{1}{K}+M+B)}{L_{\alpha }\bigg [\frac{K_{1}(\alpha )}{s}+K_{0}(\alpha )\bigg ]s^{\alpha }}\bigg ]}}\nonumber \\ & \quad + \bigg [\frac{\bigg (1-e^{-s}\bigg )\text {Gr}s^{-2}}{L_{\alpha }(\text {Pr}s+\text {Pr}Q)-(B+s+K^{-1}+M)}\bigg ] \bigg [1+\frac{\text {Sc}Du(\text {Pr}s+\text {Pr}R)}{(s+R)- (\text {Sc}s+\text {Sc}Q)\text {Pr}}\bigg ] \nonumber \\ & \quad \times \bigg [e^{-x\sqrt{\bigg [\frac{(s+M+B+\frac{1}{K})}{L_{\alpha }s^{\alpha }\bigg [\frac{K_{1}(\alpha )}{s}+K_{0}(\alpha )\bigg ]}\bigg ]}}- e^{-x\sqrt{\bigg [\frac{(s+Q)s^{-\alpha }\text {Pr}}{\bigg (\frac{K_{1}(\alpha )}{s}+K_{0}(\alpha )\bigg )}\bigg ]}}\bigg ]\nonumber \\ & \quad + \bigg (\frac{(1-e^{-s})s^{-2}}{L_{\alpha }(\text {Sc}s+R\text {Sc})-(B+M+s+K^{-1})}\bigg ) \bigg [\text {Gm}-\frac{\text {Sc}\text {Gr}\text {Pr}(Dus+DuR)}{(\text {Sc}s+\text {Sc}R)- (s+Q)\text {Pr}}\bigg ]\nonumber \\ & \quad \times \bigg (e^{-x\sqrt{\bigg [\frac{(M+s+K^{-1}+B)}{L_{\alpha }\bigg [\frac{K_{1}(\alpha )}{s}-K_{0}(\alpha )\bigg ]s^{\alpha }}\bigg ]}}- e^{-x\sqrt{\bigg [\frac{(R+s)\text {Sc}s^{-\alpha }}{\bigg (\frac{K_{1}(\alpha )}{s}+K_{0}(\alpha )\bigg )}\bigg ]}}\bigg ). \end{aligned}$$

The inverse of Eq. (35) is taken with the help of algorithm^48,49^.

### Sherwood number

Sh is given by from Eq. (26),

36 $$\begin{aligned} Sh=-L^{-1}\bigg [-\bigg (s^{-2}-s^{-2}e^{-s}\bigg )\sqrt{\frac{\text {Sc}(s+R)}{\bigg (\frac{K_{1}(\alpha )}{s}+K_{0}(\alpha )\bigg )s^{\alpha }}}\bigg ]. \end{aligned}$$

### Nusselt number

From Eq. (30),

37 $$\begin{aligned} Nu & =-L^{-1}\bigg [\frac{1-e^{-s}}{-s^{2}}\sqrt{\frac{(\text {Pr}s+Q\text {Pr})}{\bigg [\frac{K_{1}(\alpha )}{s}+K_{0}(\alpha )\bigg ]s^{\alpha }}}\nonumber \\ & \quad + \bigg (\frac{s^{-2}(Du-Due^{-s})\text {Sc}(s+R)\text {Pr}}{(s+R)\text {Sc}- (s\text {Pr}+Q\text {Pr})}\bigg )\bigg ( \sqrt{\frac{s\text {Pr}+Q\text {Pr}}{\bigg [\frac{K_{1}(\alpha )}{s}+K_{0}(\alpha )\bigg ]s^{\alpha }}} \nonumber \\ & \quad - \sqrt{\frac{s\text {Sc}+R\text {Sc}}{\bigg [\frac{K_{1}(\alpha )}{s}+K_{0}(\alpha )\bigg ]s^{\alpha }}}\bigg )\bigg ]. \end{aligned}$$

### Skin friction

Nu is given by from Eq. (34),

38 $$\begin{aligned} \tau= & -L^{-1}\bigg [\frac{-1}{s-a}\sqrt{\bigg [\frac{(s+B+\frac{1}{K}+M)}{L_{\alpha }\bigg [\frac{K_{1}(\alpha )}{s}+K_{0}(\alpha )\bigg ]s^{\alpha }}\bigg ]}\nonumber \\ & + \bigg [\frac{-\bigg (1-e^{-s}\bigg )\text {Gr}s^{-2}}{L_{\alpha }(\text {Pr}s+\text {Pr}Q)-(s+K^{-1}+M+B)}\bigg ] \bigg [1+\frac{\text {Sc}\text {Pr}(Dus+DuR)}{(\text {Sc}s+\text {Sc}R)- (\text {Pr}s+Q\text {Pr})}\bigg ] \nonumber \\ & \times \bigg [\sqrt{\bigg [\frac{(s+M+B+\frac{1}{K})}{L_{\alpha }s^{\alpha }\bigg [\frac{K_{1}(\alpha )}{s}+K_{0}(\alpha )\bigg ]}\bigg ]}- \sqrt{\bigg [\frac{(\text {Pr}s+\text {Pr}Q)s^{-\alpha }}{\bigg (\frac{K_{1}(\alpha )}{s}+K_{0}(\alpha )\bigg )}\bigg ]}\bigg ]\nonumber \\ & + \bigg (-\frac{s^{-2}(1-e^{-s})}{L_{\alpha }(\text {Sc}s+\text {Sc}R)-(K^{-1}+s+M+B)}\bigg ) \bigg [\text {Gm}-\frac{\text {Gr}\text {Pr}(Du\text {Sc}s+Du\text {Sc}R)}{(s+R)\text {Sc}- (\text {Pr}s+\text {Pr}Q)}\bigg ]\nonumber \\ & \times \bigg [\sqrt{\bigg [\frac{(M+s+B+K^{-1})}{L_{\alpha }\bigg [\frac{K_{1}(\alpha )}{s}-K_{0}(\alpha )\bigg ]s^{\alpha }}\bigg ]}- \sqrt{\bigg [\frac{(R+s)\text {Sc}}{\bigg (\frac{K_{1}(\alpha )}{s}+K_{0}(\alpha )\bigg )s^{1-\alpha }}\bigg ]}\bigg ]\bigg ]. \end{aligned}$$

## Discussions

A semi-analytical solution including a temperature gradient and combined concentration across a plate for Brinkman fluid is achieved. The fractional fractional derivative is utilized to solve the generalized model. Plots of the temperature profile, velocity profile, and concentration profile are made for various parameters. The parametric values in the study are chosen based on their relevance to the physical phenomena being modeled, such as natural convection near a vertical plate. These values typically correspond to specific conditions like fluid properties (e.g., Prandtl number), geometric dimensions, and temperature differences that reflect real-world applications. By selecting these values, the study aims to simulate realistic scenarios and capture the essential characteristics of the heat transfer and flow behavior under isothermal conditions.

**Fig. 1:**
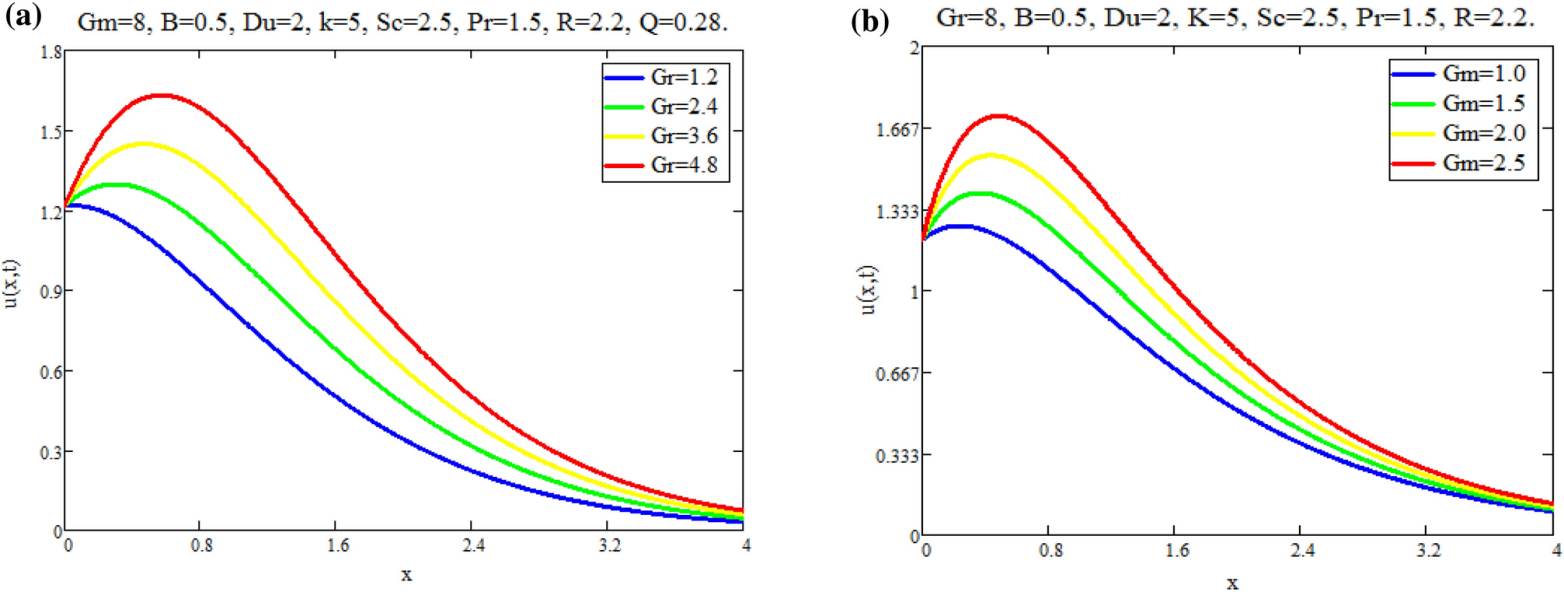
(**a**) Velocity profile u(x, t) for different values of Gr. (**b**) Velocity profile u(x, t) for different values of Gm.

**Fig. 2:**
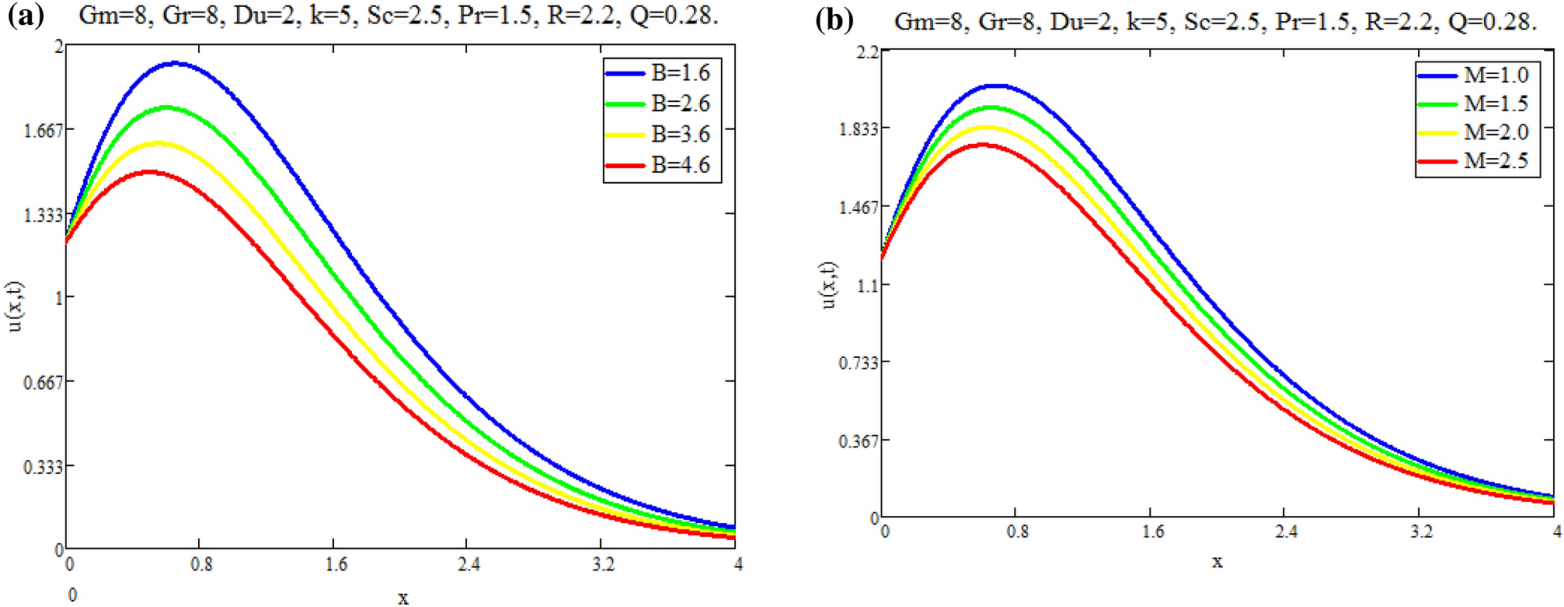
(**a**) Velocity profile u(x, t) for different values of B. (**b**) Velocity profile u(x, t) for different values of M.

**Fig. 3:**
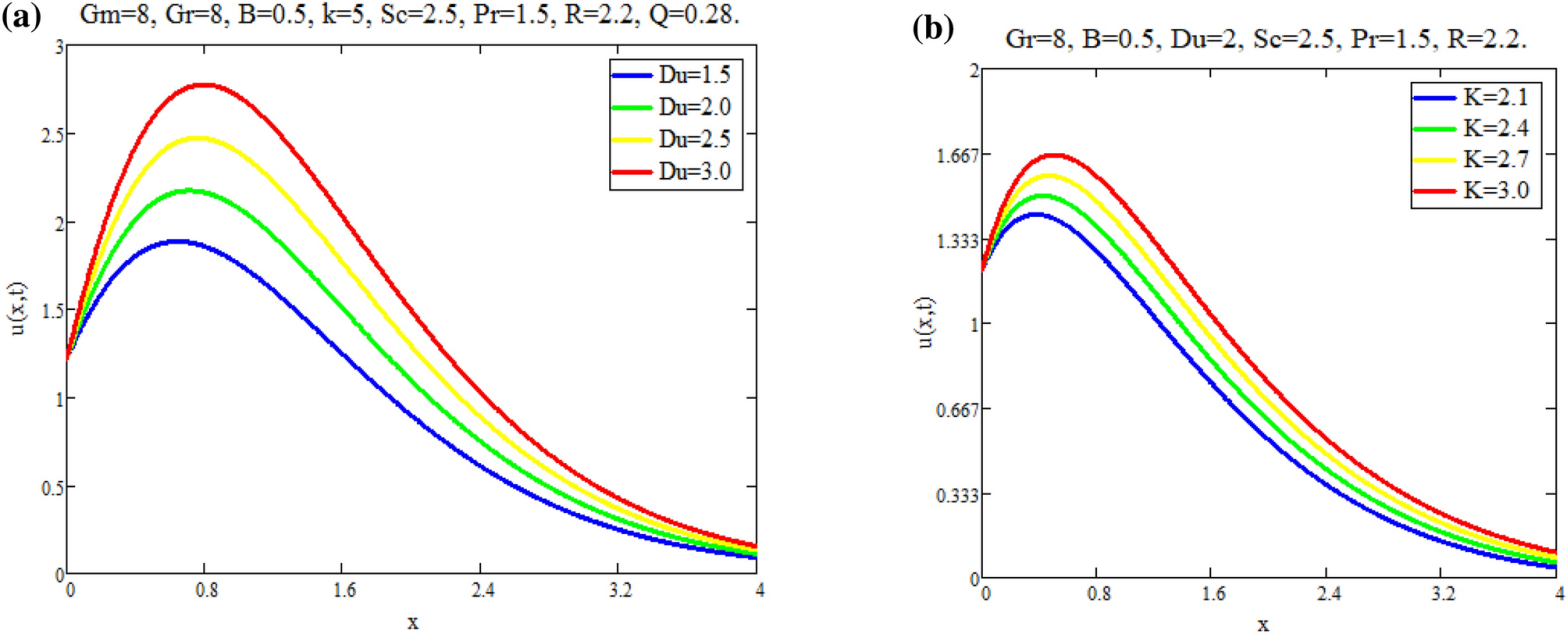
(**a**) Velocity profile u(x, t) for different values of Du. (**b**) Velocity profile u(x, t) for different values of K.

**Fig. 4:**
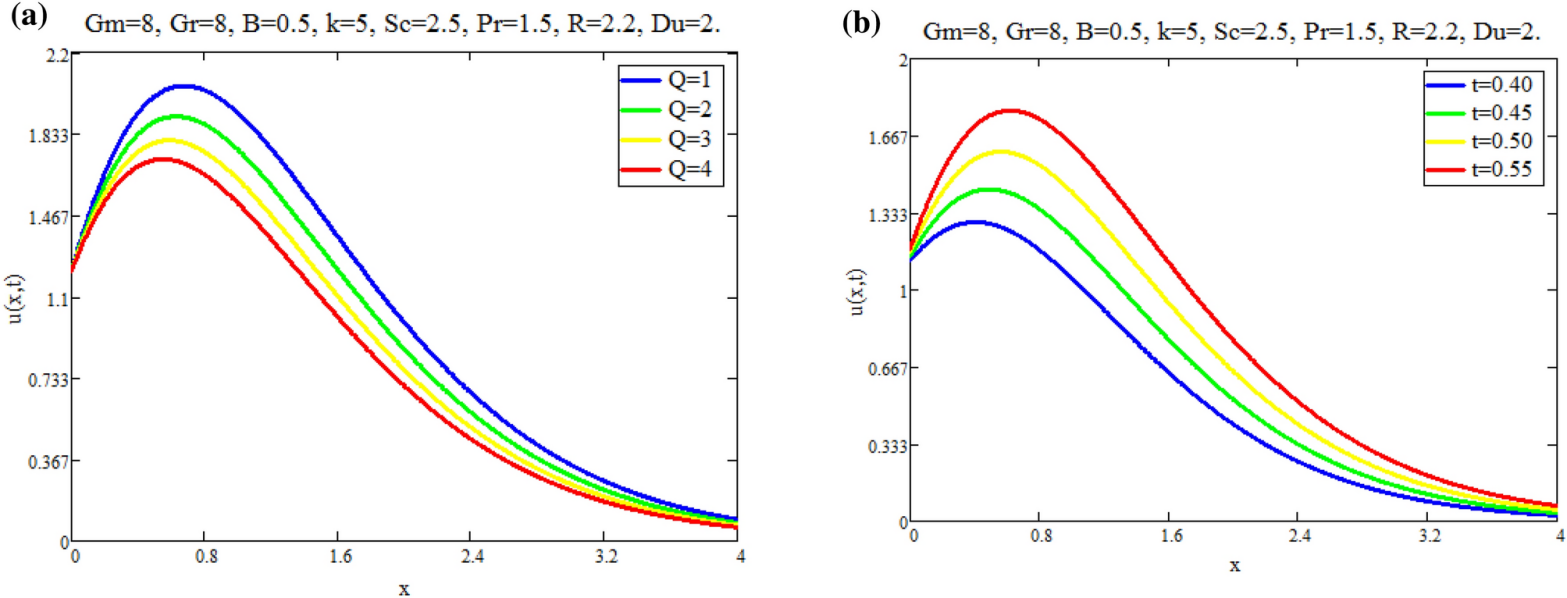
(**a**) Velocity profile u(x, t) for different values of Q. (**b**) Velocity profile u(x, t) for different values of t.

The impact of $\text {Gr}$ on velocity distribution *u*(*x*, *t*) is seen in Fig. 1a. It can be shown from this graph that the velocity distribution and $\text {Gr}$ are exactly related. In physical terms, $\text {Gr}$ represents the relationship between buoyant force and viscous force. Consequently, buoyancy force rises with a increase in $\text {Gr}$ values, raising the magnitude of velocity of fluid. The effect of $\text {Gm}$ on distribution of velocity *u*(*x*, *t*) is seen in Fig. 1b. The advanced buoyancy force becomed dominent for larger values of *Gm* which rais the fluid motion as depicted in Fig. 2a represents the influence of various values of $\text {B}$ on fluid which repots that fluid motion retards for larger values of *B*. *M*’s behavior in fluid motion is seen in Fig. 2b. As seen by the graph, the fluid layer’s speed decreases as *M* increases. Within the solution, the Lorentz force physically generates low resistance, increasing the thickness or breadth of the momentum barrier layer and causing the u(x,t) to decay. Figure 3a shows the effect of Du on fluid mobility. Figures show that the fluid layers rise as the value of Du accelerates. The effect of *K* on fluid is seen in Fig. 3b. The velocity of fluid is enhanced with accelerating the values of *K*, due to weaker viscous drag of media of porous. The behavior of *Q* with respect to fluid motion is seen in Fig. 4a. As Fig. shows, rising values of *Q* lower the magnitude of fluid. Physically speaking, thermal conductivity will decrease as the heat absorption parameter values rise. The fluid motion is diminished by the strong attraction between fluid particles. Figure 4b represents the effect of time *t* on velocity fields. It is noted that motion of fluid has directly proportional with time.

**Fig. 5:**
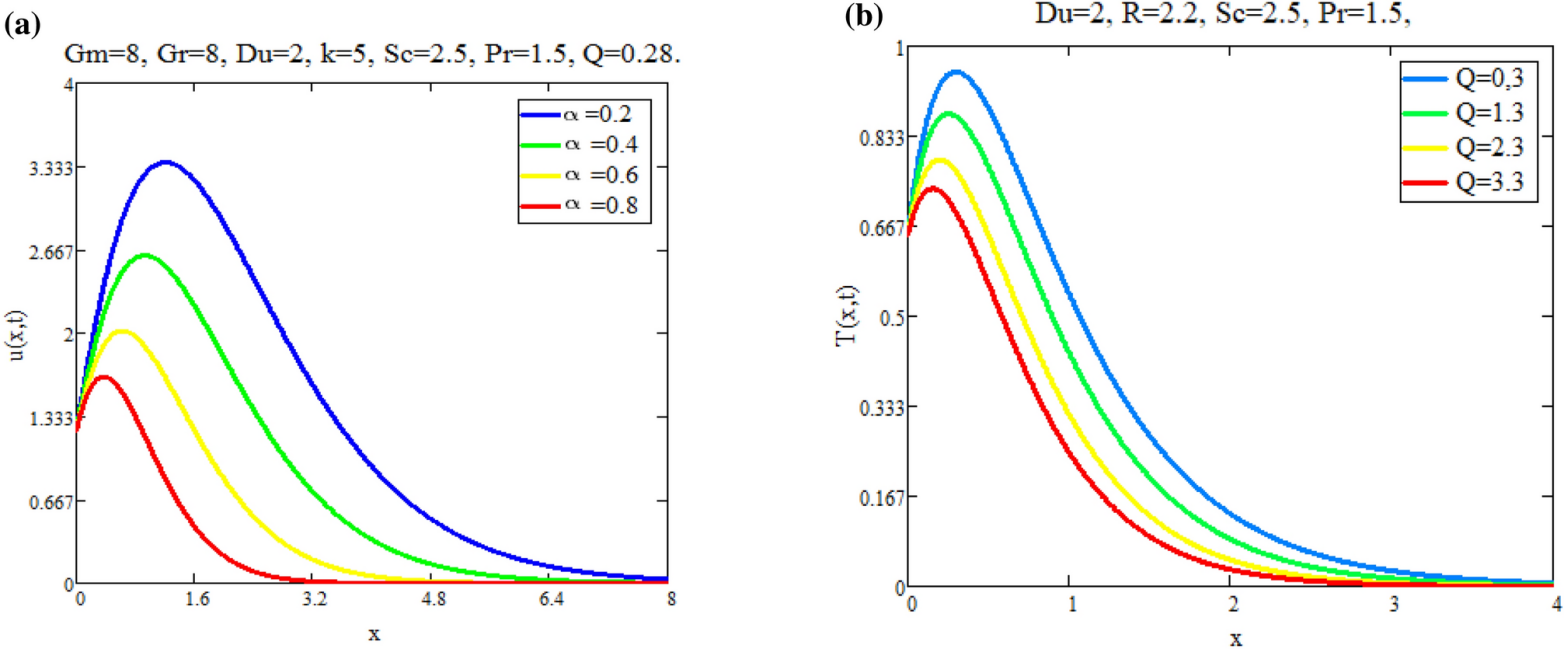
(**a**) Velocity profile u(x, t) for different values of α. (**b**) Temperature profile T(x, t) for different values of Q.

**Fig. 6:**
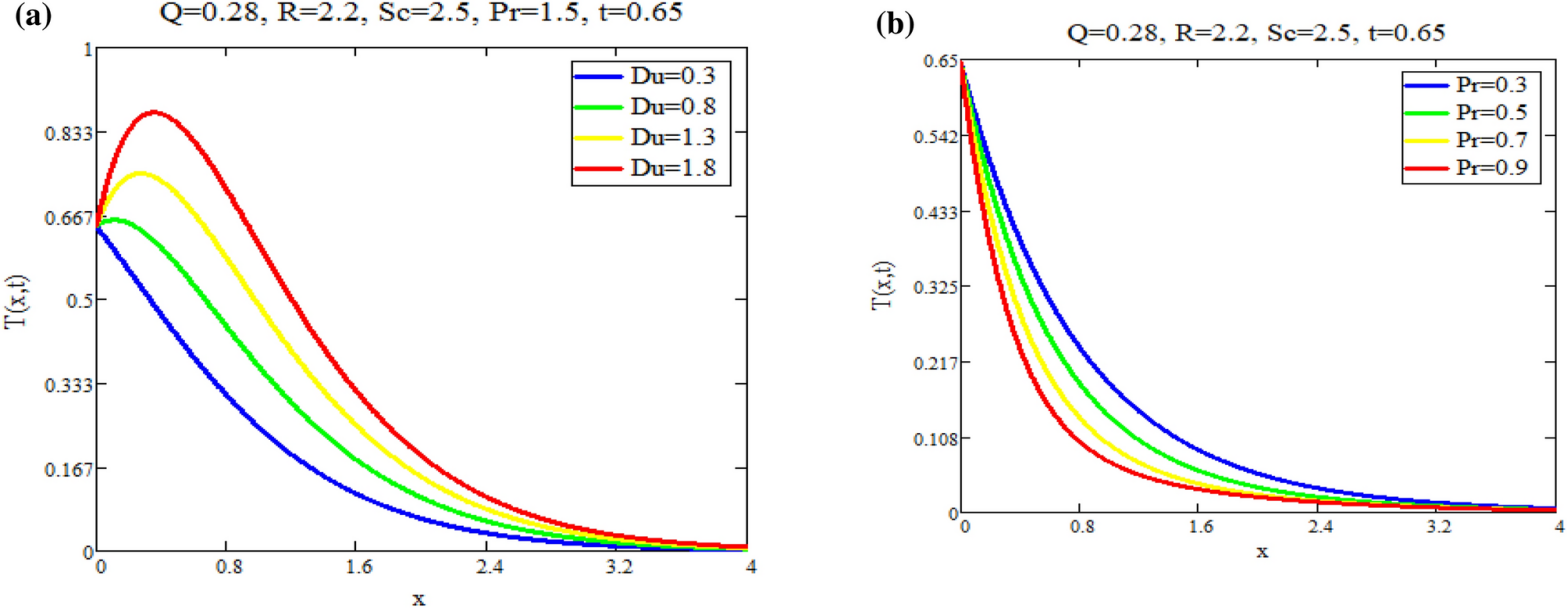
(**a**) Temperature profile T(x, t) for different values of Du. (**b**) Temperature profile T(x, t) for different values of Pr.

**Fig. 7:**
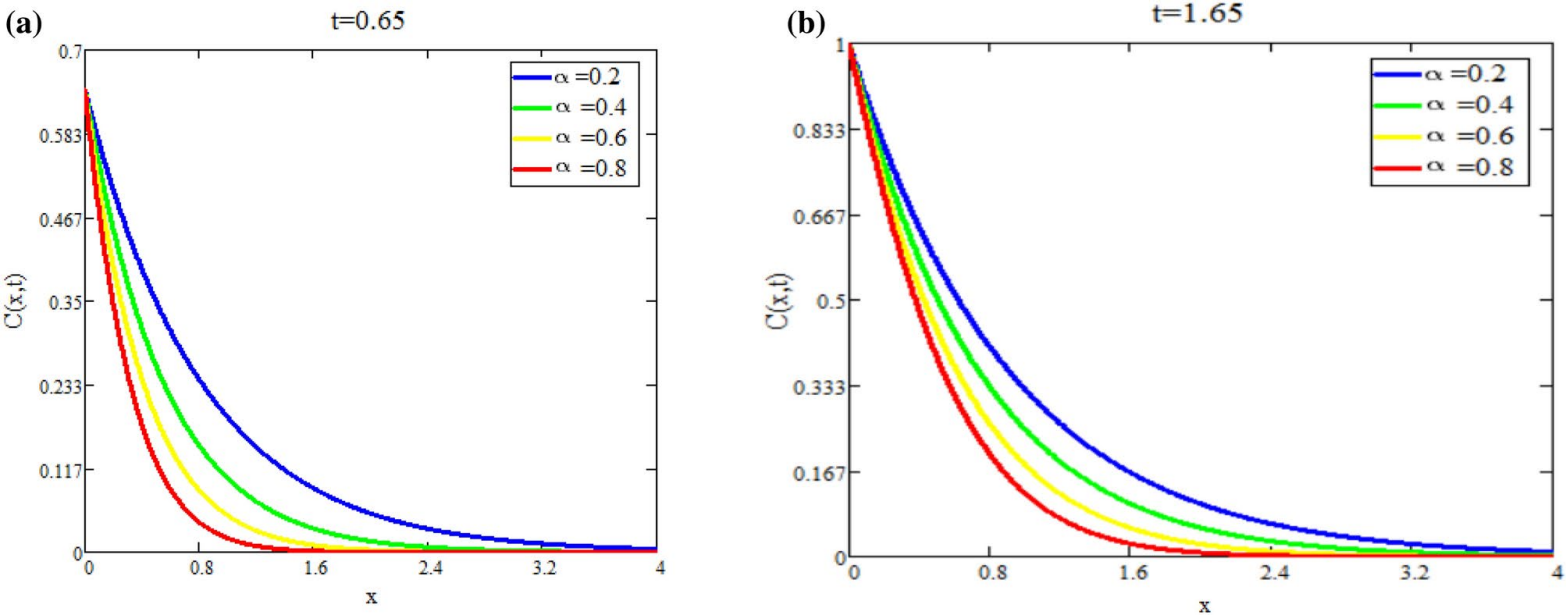
(**a**) Concentration profile C(x, t) for different values of α. (**b**) Concentration profile C(x, t) for different values of α for increasing time.

**Fig. 8:**
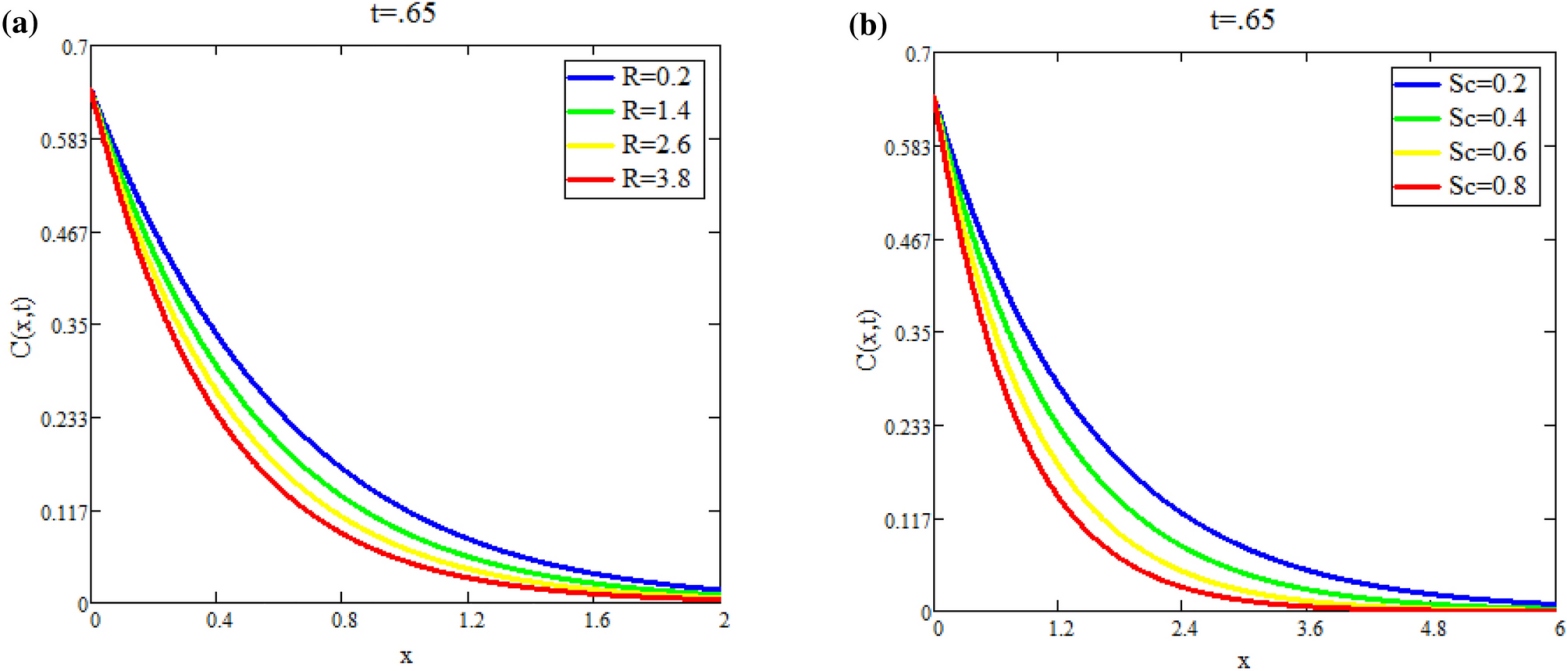
(**a**) Concentration profile C(x, t) for different values of R. (**b**) Concentration profile C(x, t) for different values of Sc.

Figure 5a shows the effect of fractional parameter on velocity fields. It is observed that motion of fluid has reverse behavior with fractional parameters. The impact of *Q* on fluid *T*(*x*, *t*) is reported in Fig. 5b. Figure 6a highlight the effect of $\text {Du}$ on fluid’s *T*(*x*, *t*) for small time $t=0.60$ which indicates that temperature of fluid rises with enhancing values of *Du*. Figure 6b shows the effect of *Pr* on temperature. The temperatue of fluid is decay down with accelerating values of Pr due to the fact that thermal conductivity have strongly effect on fluid for larger values of Pr. The impact of the fractional parameter on the concentration profile is seen in Fig. 7a,b. Figure 8a depicts the behavior of $\text {R}$ on *C*(*x*, *t*). As the graph illustrates, the concentration level increases as $\text {R}$ decreases. The impact of $\text {Sc}$ on *C*(*x*, *t*) is seen in Fig. 8b. As seen in the image, the mass level rises with decay values of $\text {Sc}$. Graph illustrate that larger values of $\text {Sc}$ raise the diffusion of molecule which decreases the concentration level.

**Fig. 9:**
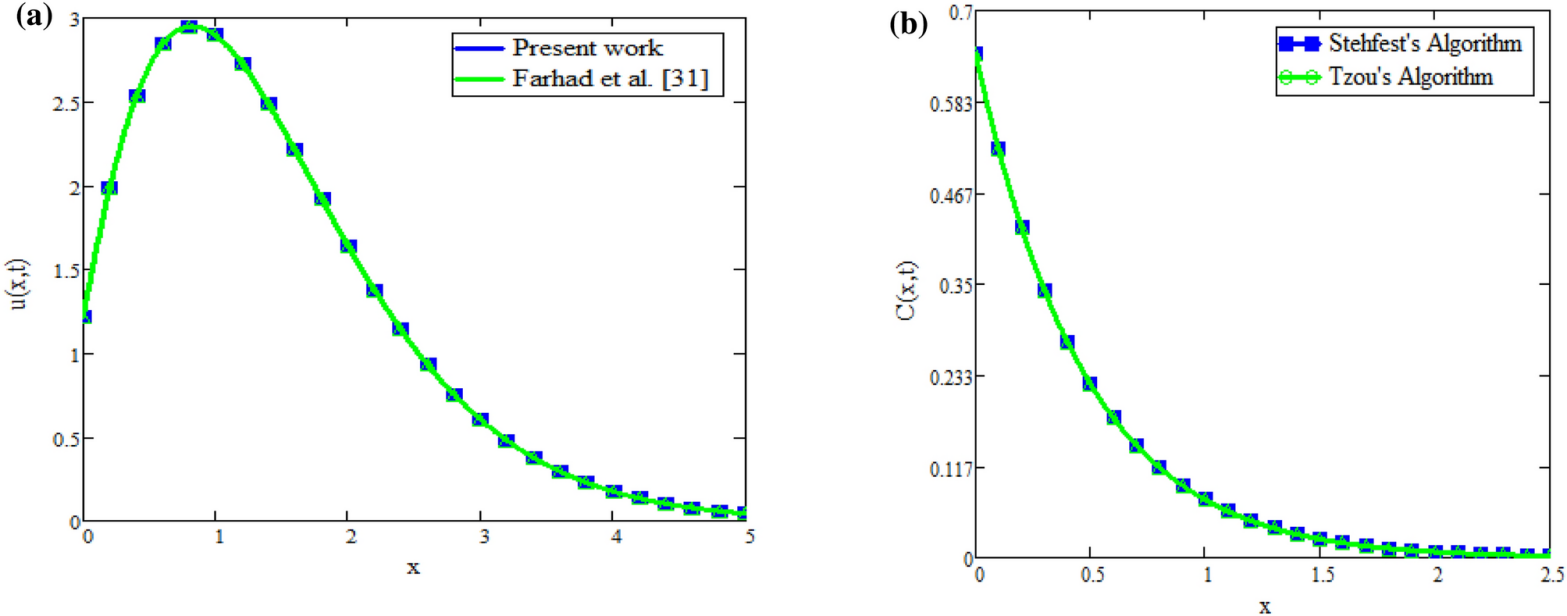
(**a**) Velocity distribution for comparison of our work with Farhad et al.^31^. (**b**) Concentration obtain by Stehfest’s and Tzou’s algorithm.

**Fig. 10:**
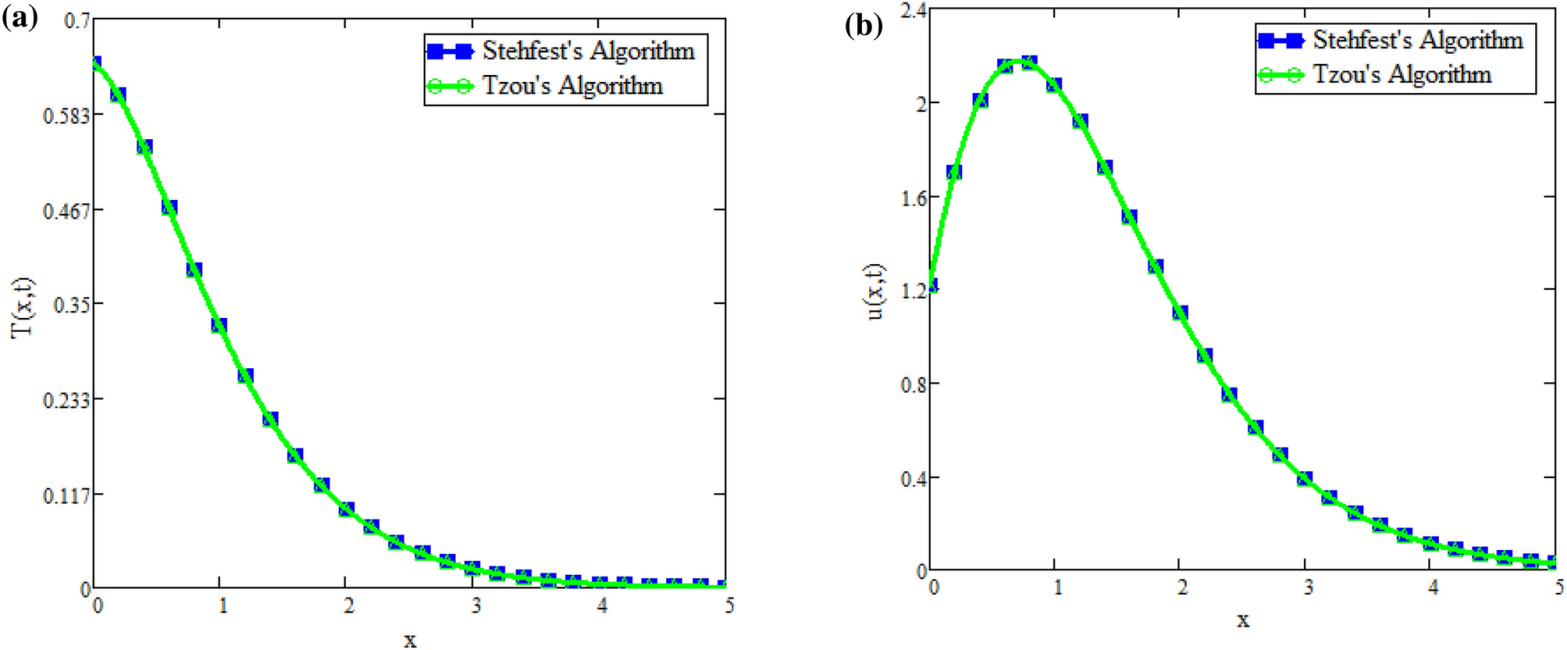
(**a**) Temperature obtain by Stehfest’s and Tzou’s algorithm. (**b**) Velocity obtain by Stehfest’s and Tzou’s algorithm.

**Fig. 11:**
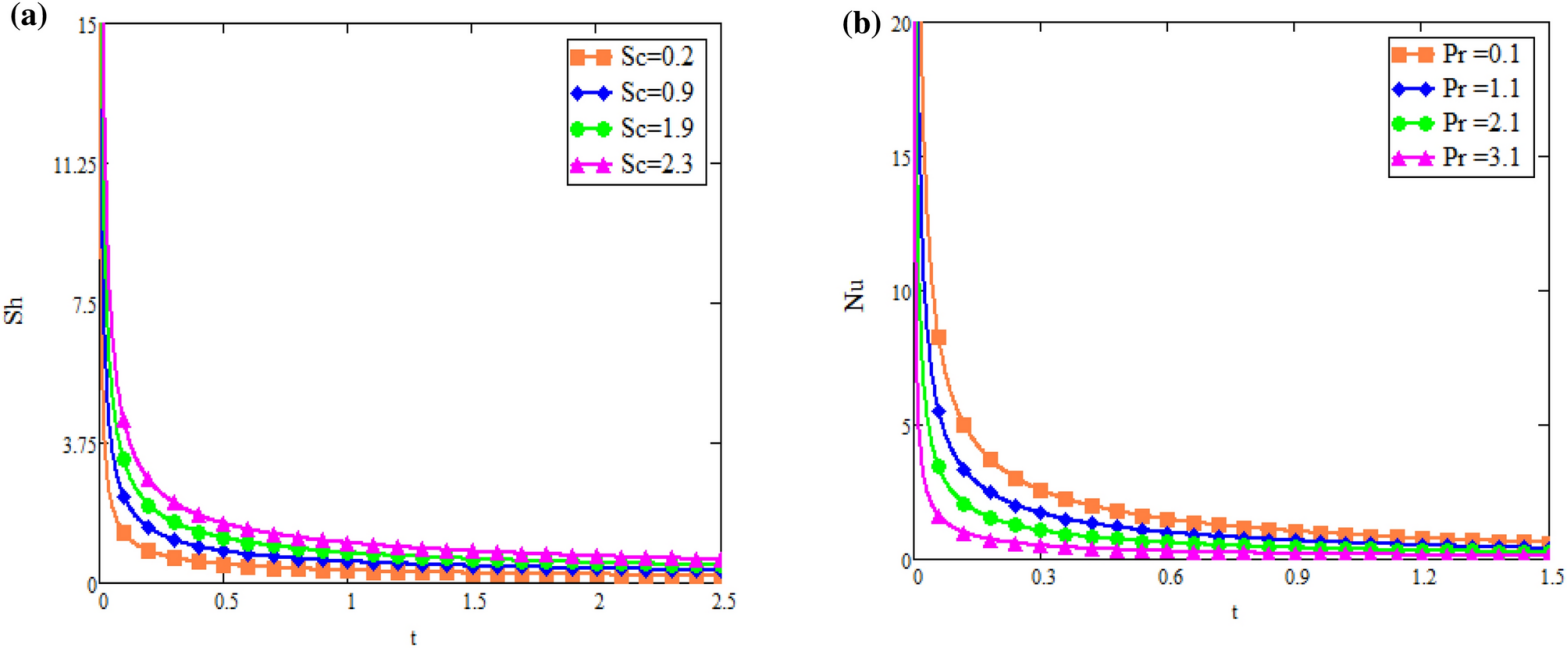
(**a**) Effect of Sc on Sherwood number. (**b**) Effect of Pr on Nusselt number.

**Fig. 12:**
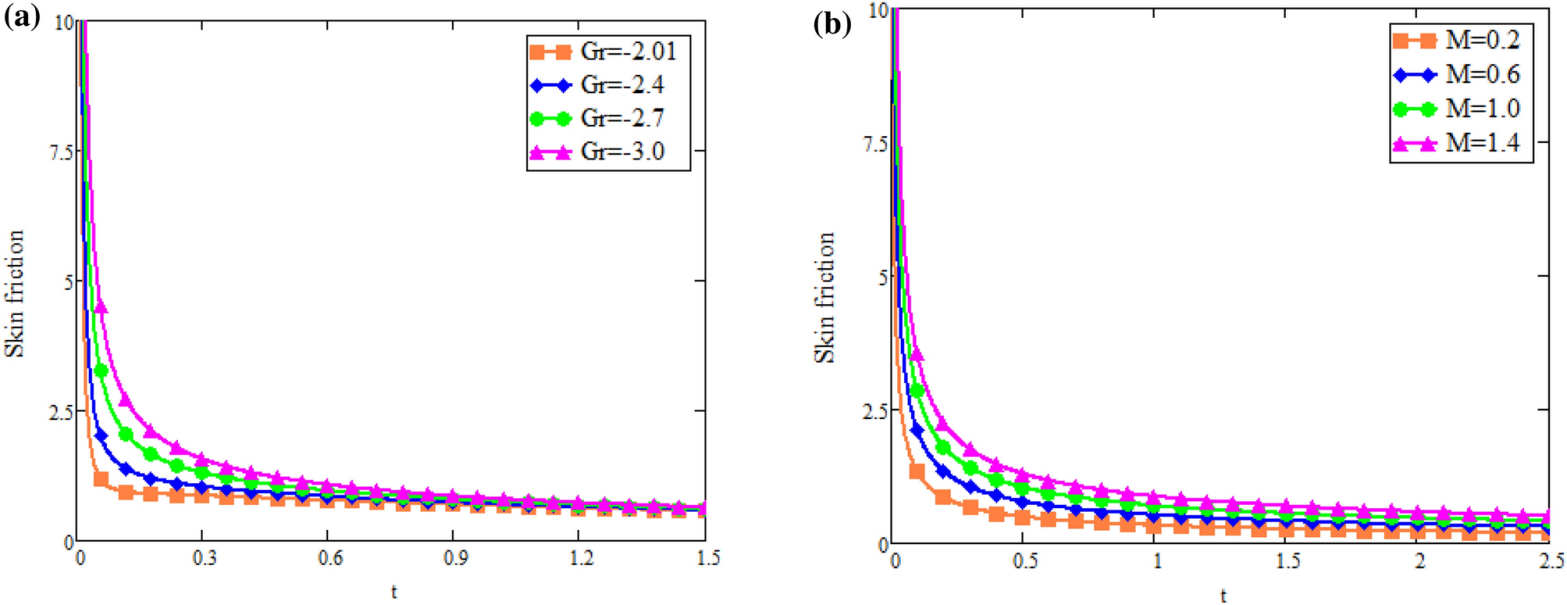
(**a**) Effect of Gr on skin friction T. (**b**) Effect of M on skin friction T.

The fractional fluid comparison with Farhad et al.^31^ is displayed in Fig. 9a. Figure illustrates how the fluid profiles are the same when fractional parameters, *Gr*, *Gm*, the Brinkman parameter, and no slip are present. This demonstrates the validity of the current study. Figures 9b and 10b illustrate the applicability of inverse techniques for temperature, velocity, and concentration profiles. Figures 11a and 12b represents the Sherwood number, Nusselt number and Skin friction respectively.

## Validation of result

**Fig. 13:**
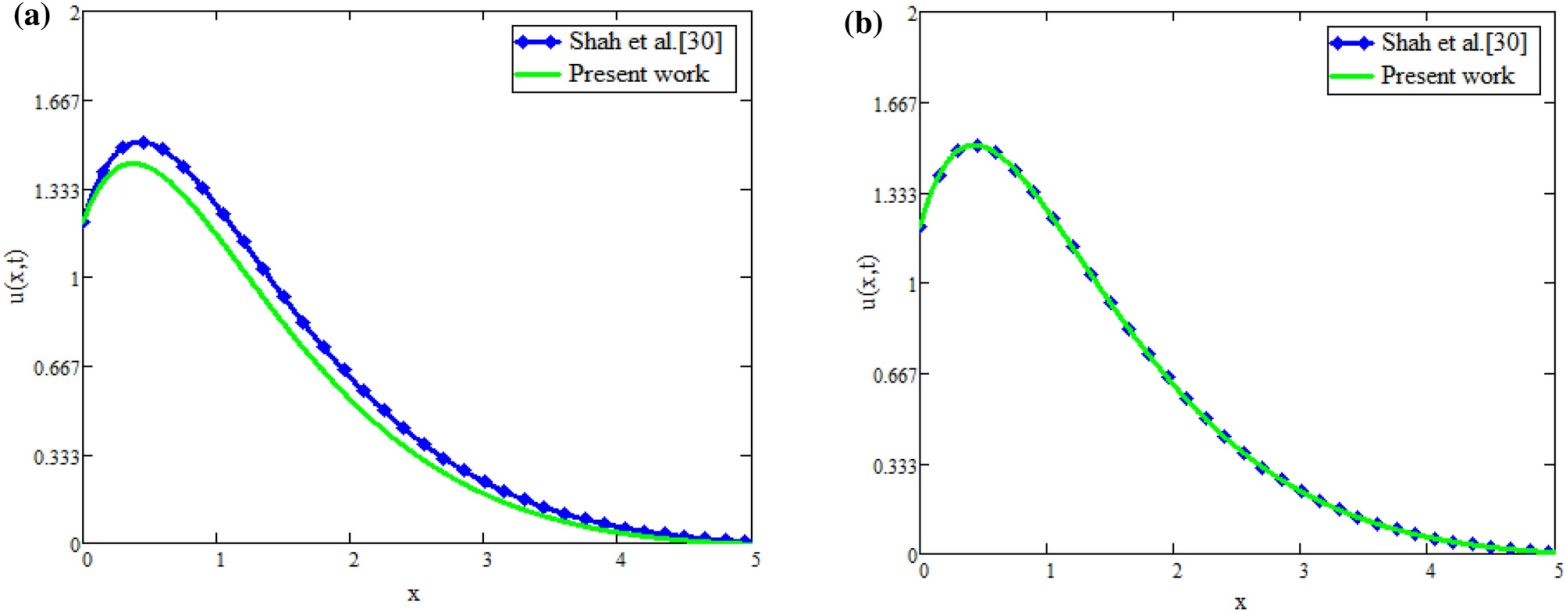
(**a**) Comparison of velocity distribution. (**b**) Comparison of velocity distribution.

This section represents the validity of present study with already published work. with the help of suitable assumption, the velocity profiles of present study are compared with Shah et al.^30^ and Farhad et al.^31^. In the absence of Brinkman parameter, heat sink parameter, diffusion effect, fractional parameter, chemical reaction, the velocity profiles of present work and Farhad et al.^31^ are adentical as shown in Fig. 9a, which shows the validity of preset work. Similarly Fig. 13a,b shows the validity of present work. Figure 13a represents that velocity profile for constant Caputo proportional derivative is smaller as compared to caputo fractional derivative, therefore constant Caputo proportional derivative is best choice to obtaind controlled velocity of fluid. Figure 13b represents that velocity profiles becomes identical for both present work and Shah et al.^30^ which gives the validity of presents work. Also the experimental validation of the model using various fractional parameter values as shown in Table 1.

**Table 1:** Validation of the model using various fractional parameter values.

Fractional parameters	Temperature with Stehfest’s	Temperature with Tzou’s	Velocity with Stehfest’s	Velocity with Tzou’s
0.0	0.72490	0.71890	0.00000	0.00000
0.2	0.55081	0.54481	0.13681	0.13681
0.4	0.41772	0.41372	0.18672	0.18672
0.6	0.31663	0.31463	0.18763	0.18763
0.8	0.24054	0.23954	0.16654	0.16654
1.0	0.18245	0.18145	0.13745	0.13745
1.2	0.13836	0.13836	0.10836	0.10836
1.4	0.10527	0.10527	0.08327	0.08327
1.6	0.08018	0.07918	0.06318	0.06318
1.8	0.06099	0.06009	0.04809	0.04809
2.0	0.04680	0.04690	0.03690	0.03690
2.2	0.03571	0.03581	0.02781	0.02781
2.4	0.02662	0.02672	0.02072	0.02072
2.6	0.01553	0.02063	0.01563	0.01563
2.8	0.01144	0.01554	0.01154	0.01154
3.0	0.00865	0.01145	0.00860	0.00860

## Experimental validation

**Fig. 14:**
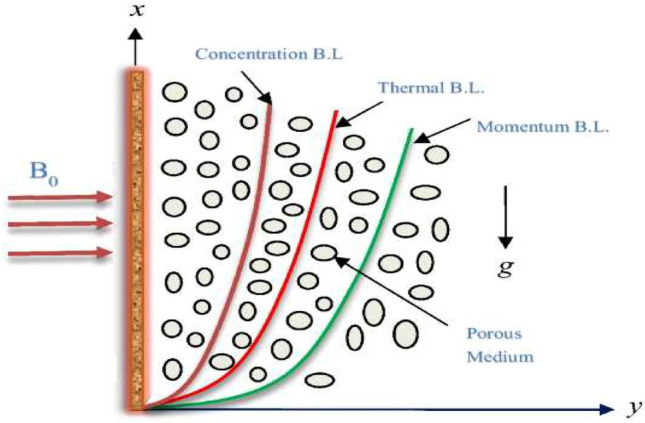
Flow geometry.

The authors developed correlations for the Nusselt number ($Nu$) and Sherwood number ($Sh$) as functions of various parameters such as Prandtl number ($Pr$) and Schmidt number ($Sc$) (Fig. 14).

To illustrate their findings, the results were presented in graphical form, typically including:

1. *Nusselt number correlations* Graphs showing the variation of $Nu$ with $Pr$ for different boundary conditions, indicating how convective heat transfer changes with varying thermal driving forces.
2. *Sherwood number correlations* Graphs depicting the relationship between $Sh$ and $Sc$, demonstrating the influence of diffusion on mass transfer rates.
3. *Skin friction coefficient correlations* Variation of $C_f$ with $Gr$ and $M$, highlighting the impact of buoyancy-driven flow and inertial effects on frictional resistance.

These graphs help visualize the performance of the thermal and mass transfer processes and validate the theoretical predictions against experimental or numerical data.

## Findings

The fluid flow problem involving free convection magnetohydrodynamics has been solved semi-analytically. Plots and discussions are shown for the various model parameters. Fractional derivatives are used to solve the model.

Summarized for this model are the following primary points:

- As the fractional parameter values increase, the velocity distribution slows down.
- The *u*(*x*, *t*) accelerates due to thermal buoyancy forces.
- As the magnetic force rises, *u*(*x*, *t*) decreases.
- The movement of fluid is speed up for accelerating $\text {Du}$.
- The smaller values of $\alpha$ accelerate the temperature of fluid *T*(*x*, *t*).
- The concentration level is decay with raising $\text {Sc}$
- The smaller values of *R* accelerate the concentration level.

## Applications

- It has wide uses in biomedical engineering due to the behavior of non newtonian fluids.
- It also uses in petroleum industry due to the behavior of heal flux, which can be used to more efficient cooling systems.
- It has wide uses in polymers processing.
- It can be applied to environmental remediation processes i.e oil pumps.

## Limitations

The present model is made with the help of generalized Fourier’s and Fick’s laws by using constant Caputo fractional derivative. However, in order to understand the comprehensive implications and scope of the study, some limitation should be addressed. The complexity of mathematical model have simplification which are necessary for the physical system. The material properties of the fluid can vary significantly. The boundary conditions for the model may not fully capture the walls of plate.

## Future scope

The model can be extend by using nanofluid properties involving concentrations, shapes and various sizes in order to capture the better behavior of real word with fluid flow. The advanced model can be developed by using entropy generation and bioconvection in the governing equations. Investigate how biochemical reactions, behavior of nanofluid, and dynamics of microorganism enhanced the reality of the model.

## Data Availability

The datasets used and/or analysed during the current study available from the corresponding author on reasonable request.

## List of symbols

$C_{w}$: Concentration at the plate [kg m$^{-3}$]
*D*: Diffusion of mass [m$^{2}$ s$^{-1}$]
$$\text {Gr}$$: Grashof number $$[\beta _{T} T_{w}]$$
$\text {Pr}$: Number of Prandtl $\bigg [\mu \frac{Cp}{k}\bigg ]$
$T_{\infty }$: Fluid temperature far away from plate [K]
$\mu$: Dynamic viscosity [kg m$^{-1}$ s$^{-1}$]
$C_{\infty }$: Fluid concentrate at long distance from the plate [kg m$^{-3}$]
$\rho$: Fluid density [kg m$^{-3}$]
*u*: Speed of fluid [ms$^{-1}$]
$\beta _{T}$: Volumetric coefficient of thermal expansion [K$^{-1}$]
*g*: gravitational acceleration [ms$^{-2}$]
$\beta _{C}$: Coefficient of volumetric expansion with species concentration [K$^{-1}$]
$T_{w}$: Fluid temperature at the plate [K]
$\beta _{C}$: Volumetric coefficient of mass expansion [m$^{3}$ kg$^{-1}$]
*q*: Variable of Laplace transform
$B_{0}$: Constant magnetic field
$$\alpha ,\beta$$: Fractional parameter
